# Clinical significance of the advanced lung cancer inflammation index in patients with limited-stage small cell lung cancer treated with chemoradiotherapy

**DOI:** 10.1038/s41598-024-61145-9

**Published:** 2024-05-06

**Authors:** Bo Mi Seo, Jiin Choi, Boksoon Chang, Bo-Guen Kim, Tai Sun Park, Hyun Lee, Ji-Yong Moon, Sang-Heon Kim, Tae-Hyung Kim, Seung-Jin Yoo, Hae Jin Park, Ho Joo Yoon, Jang Won Sohn, Seung Hyeun Lee, Dong Won Park

**Affiliations:** 1 Department of Internal Medicine, Hanyang University College of Medicine, 222-1 Wangsimni-ro, Seongdong-gu, Seoul, 04763 Republic of Korea; 2 Office of Hospital Information, Seoul National University Hospital, Seoul, South Korea; 3 Division of Pulmonary, Allergy, and Critical Care Medicine, Department of Internal Medicine, Kyung Hee University College of Medicine, Kyungheedae-ro 23, Dongdaemun-gu, Seoul, 02447 Republic of Korea; 4 Department of Radiology, Hanyang University College of Medicine, Seoul, South Korea; 5 Department of Radiation Oncology, Hanyang University College of Medicine, Seoul, South Korea

**Keywords:** Cancer, Lung cancer, Tumour biomarkers

## Abstract

The aim of the study was to investigate the prognostic significance of the advanced lung cancer inflammation index (ALI) in patients with limited-stage small-cell lung cancer (LS-SCLC) undergoing definite chemo-radiotherapy (CRT). We included 87 patients with LS-SCLC from South Korea, treated between 2005 and 2019 with definite CRT. ALI was calculated using body mass index, serum albumin, and neutrophil–lymphocyte ratio. We categorized 38 patients into the high ALI group (ALI ≥ 44.3) and 48 into the low ALI group (ALI < 44.3). Patients in the high ALI group exhibited longer overall survival (OS) than patients in the low ALI group. In multivariate analysis, prophylactic cranial irradiation (hazard ratio [HR] = 0.366, 95% confidence interval [CI] 0.20–0.66, P = 0.0008), and high ALI (HR = 0.475, 95% CI 0.27–0.84, P = 0.0103) were identified as independent prognostic factors for predicting better OS. Notably, a high ALI score was particularly indicative of longer survival in patients treated with the combination of etoposide and cisplatin. In conclusion, this study demonstrated that a high pretreatment ALI was significantly associated with better OS in patients with LS-SCLC undergoing definite CRT. This suggests that ALI could be a useful tool for predicting prognosis and guiding chemotherapy regimen selections in clinical practice for LS-SCLC.

## Introduction

Lung cancer remains a leading cause of death world-wide, holding the highest mortality rate among all cancers in South Korea^1^. Small cell lung cancer (SCLC) constitutes approximately about 15% of all lung cancer types, characterized by aggressive features such as rapid doubling time, a high growth fraction, and early extensive metastasis^2^. Prior to treatment, approximately 30–40% of SCLC patients present with limited-stage small cell lung cancer (LS-SCLC), qualifying them as suitable candidates for radiotherapy. The standard treatment for LS-SCLC involves concurrent chemotherapy and thoracic radiotherapy (TRT). While LS-SCLC exhibits high sensitivity to chemotherapy and radiotherapy, it is prone to relapse, with median survival ranging 15 to 20 months, from the time of diagnosis^3^. Therefore, identifying accurate prognostic indicators for LS-SCLC is crucial.

Various clinical parameters, including cancer staging, performance stage, age, and smoking status, have been suggested to be related to prognosis^4,5^. Notably, inflammation has emerged as a hallmark characteristic in cancer development and metastasis^6^. Various laboratory markers of systemic inflammation, such as C-reactive protein (CRP), albumin, and lactate dehydrogenase (LDH) levels, have been proposed for prognostication. The neutrophil-to-lymphocyte ratio (NLR) and platelet-to-lymphocyte ratio (PLR) have demonstrated prognostic value in various malignancies, such as gastric cancer, head and neck cancer, melanoma, and lung cancer^7–11^. Recently, a new prognostic marker, the advanced lung cancer inflammation index (ALI), based on height, weight, serum albumin, and NLR, has been suggested for patients with lung cancer^12–14^. In SCLC research, earlier studies have proposed ALI as a prognostic indicator^13,15^, but the limited sample sizes of patients with LS-SCLC have hindered comprehensive research.

This study aimed to investigate the prognostic significance of ALI in the peripheral blood of patients with LS-SCLC undergoing chemoradiotherapy (CRT), focusing on overall survival (OS). Additionally, we aimed to identify other factors that may modify the prognostic utility of ALI.

## Results

In total of 87 patients with LS-SCLC were analyzed, encompassing comprehensive clinical information and baseline laboratory parameters. The baseline characteristics of the study population are summarized in Table 1. The mean age of the study population was 64.7 (SD, 8.9) years, which included 75 (86.2%) male patients. A significant majority, including 85 (97.7%) patients were ever-smokers. The performance status (PS) was generally good, with 43 (49.4%) patients scoring 0, 40 (46%) scoring 1, and only 4 (4.6%) scoring 2. Underlying comorbidities included chronic obstructive pulmonary disease in 42 (48.3%), diabetes mellitus in 30 (34.5%), hypertension in 36 (41.4%), and chronic kidney disease (CKD) in 17 (19.5%) patients. Most patients received platinum-based chemotherapy concurrently during the course of RT, with 11 (12.6%) patients receiving RT sequentially. In terms of chemotherapeutic regimen, 83 (95.4%) patients received etoposide-based combination chemotherapy, while the remaining four (4.6%) received irinotecan-based combination chemotherapy. Consolidation chemotherapy was administered in 30 (40.2%) patients and prophylactic cranial irradiation (PCI) was performed in 35 (34.5%) patients.

**Table 1:** Baseline characteristics of all patients according to advanced lung cancer inflammation index (ALI) at the time of diagnosis.

Variables	All patients (N = 87)	ALI < 44.3 (N = 48)	ALI ≥ 44.3 (N = 38)	P-values
Sex				0.6151
Male	75 (86.2)	40 (16.7)	34 (89.5)	
Female	12 (13.8)	8 (83.3)	4 (10.5)	
Age, years (mean ± SD)	64.7 ± 8.9	65.1 ± 10.1	64.4 ± 7.3	0.7437
BMI, kg/m^2^ (mean ± SD)	23.6 ± 3.3	22.7 ± 3.0	24.8 ± 3.3	0.0019
Smoking, ever-smoker	85 (97.7)	47 (97.9)	37 (97.4)	1.0000
Years of initiation for radiotherapy				0.8914
2005–2009	14 (16.1)	7 (14.6)	7 (18.4)	
2010–2014	28 (32.2)	16 (33.3)	12 (31.6)	
2015–2019	45 (51.7)	25 (52.1)	19 (50.0)	
Radiotherapy dose (Gy)				1.0000
45 ≤ < 60	42 (48.3)	23 (47.9)	19 (50.0)	
≥ 60	45 (51.7)	25 (52.1)	19 (50.0)	
ECOG PS				0.7616
0	43 (49.4)	23 (47.9)	20 (52.6)	
1	40 (46.0)	22 (45.8)	17 (44.8)	
2	4 (4.6)	3 (6.3)	1 (2.6)	
COPD	42 (48.3)	25 (52.1)	16 (42.1)	0.4823
Diabetes	30 (34.5)	19 (39.6)	11 (28.9)	0.4237
Hypertension	36 (41.4)	18 (37.5)	17 (44.7)	0.6474
Chronic kidney disease	17 (19.5%)	11 (22.9%)	6 (15.8%)	0.5812
TNM stage				0.1338
I	4 (4.6)	1 (2.1)	3 (7.9)	
II	12 (13.8)	4 (8.3)	8 (21.1)	
IIIA	30 (34.5)	16 (33.3)	13 (34.2)	
IIIB/IIIC	41 (47.1)	27 (56.3)	14 (36.8)	
Schedule of radiotherapy				1.0000
Sequential	11 (12.6)	6 (12.5)	5 (13.2)	
Concurrent	76 (87.4)	42 (87.5)	33 (86.8)	
Chemotherapy regimen				0.3008
Etoposide + cisplatin	61 (70.1)	30 (62.5)	30 (78.9)	
Etoposide + carboplatin	22 (25.3)	15 (31.2)	7 (18.4)	
Others*	4 (4.6)	3 (6.2)	1 (2.6)	
Prophylactic cranial irradiation	35 (40.2)	18 (37.5)	17 (44.7)	0.6474
Consolidation chemotherapy	30 (34.5)	13 (27.1)	16 (42.1)	0.2173
Laboratory findings (mean ± SD)				
WBC	7487.9 ± 1963.9	7707.3 ± 2124.9	7157.9 ± 1716.3	0.1993
ALC	1901.8 ± 680.4	1555.9 ± 541.0	2346.1 ± 586.6	< 0.0001
ANC	4619.2 ± 1664.8	5114.1 ± 1809.3	3928.1 ± 1142.9	0.0006
NLR	2.8 ± 1.6	3.7 ± 1.7	1.7 ± 0.4	< 0.0001
Albumin (n=86)	4.1 ± 0.4	4.0 ± 0.4	4.2 ± 0.3	0.0430

*Others included combining regimens including irinotecan and cisplatin. SD: standard deviation; BMI: body mass index; ECOG PS: Eastern Cooperative Oncology Group Performance Status; WBC: white blood cell; ALC: absolute lymphocyte count; ANC: absolute neutrophil count; NLR: neutrophil to lymphocyte ratio.

**Figure 1:**
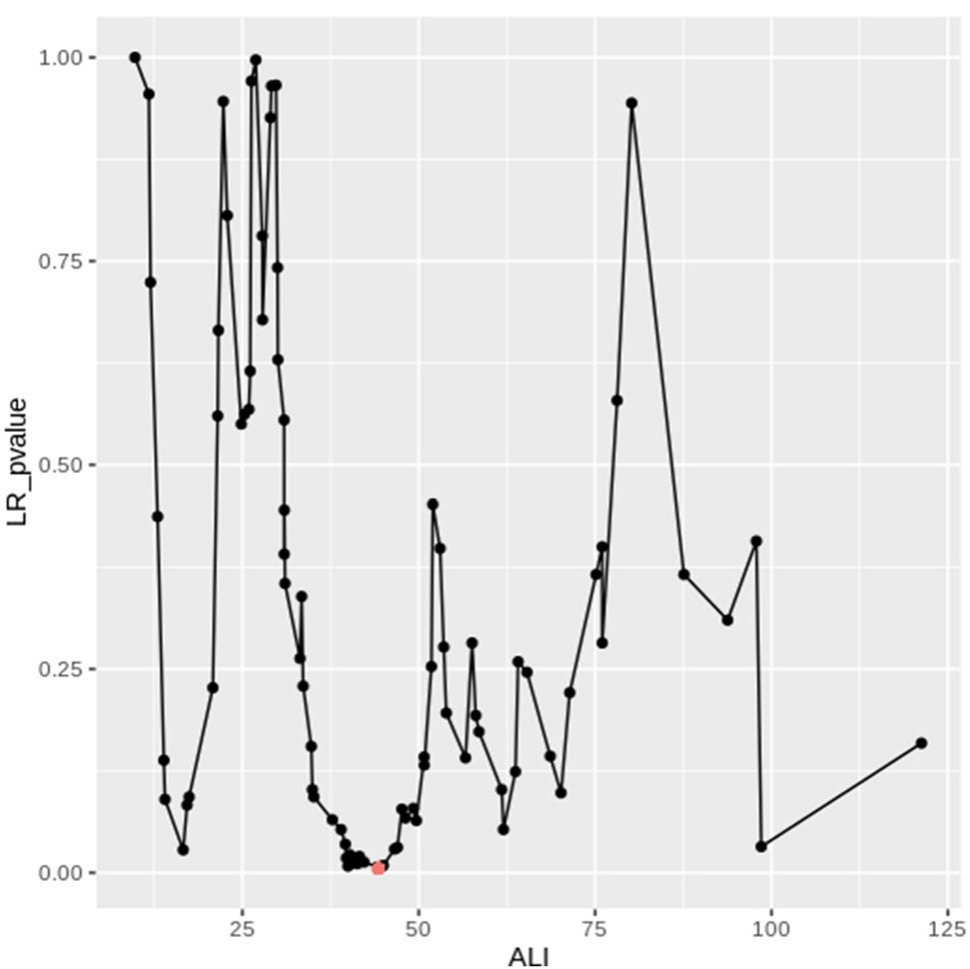
Hazard ratio for overall survival based on the cutoff-point for the advanced lung cancer inflammation index in patients with limited-stage small-cell lung cancer. The vertical line designates the optimal cutoff-point with the most significant (Log-Rank Test) split. The plot is generated using the Biostatistical Tool, Cutoff Finder.

Using the biostatistical tool Cutoff Finder^16^, the optimal cutoff point of ALI for layering OS in LS-SCLC was determined to be 44.3 (Fig. 1). Based on this cutoff value, excluding one patient whose albumin level was not measured, patients were divided into two groups: ALI < 44.3 (n = 48) and ≥ 44.3 (n = 38). Both groups demonstrated no statistical differences in terms of sex, age, smoking history, PS, underlying diseases, TNM stage, radiation dosage, schedule of radiotherapy, chemotherapy regimen, and PCI.

**Figure 2:**
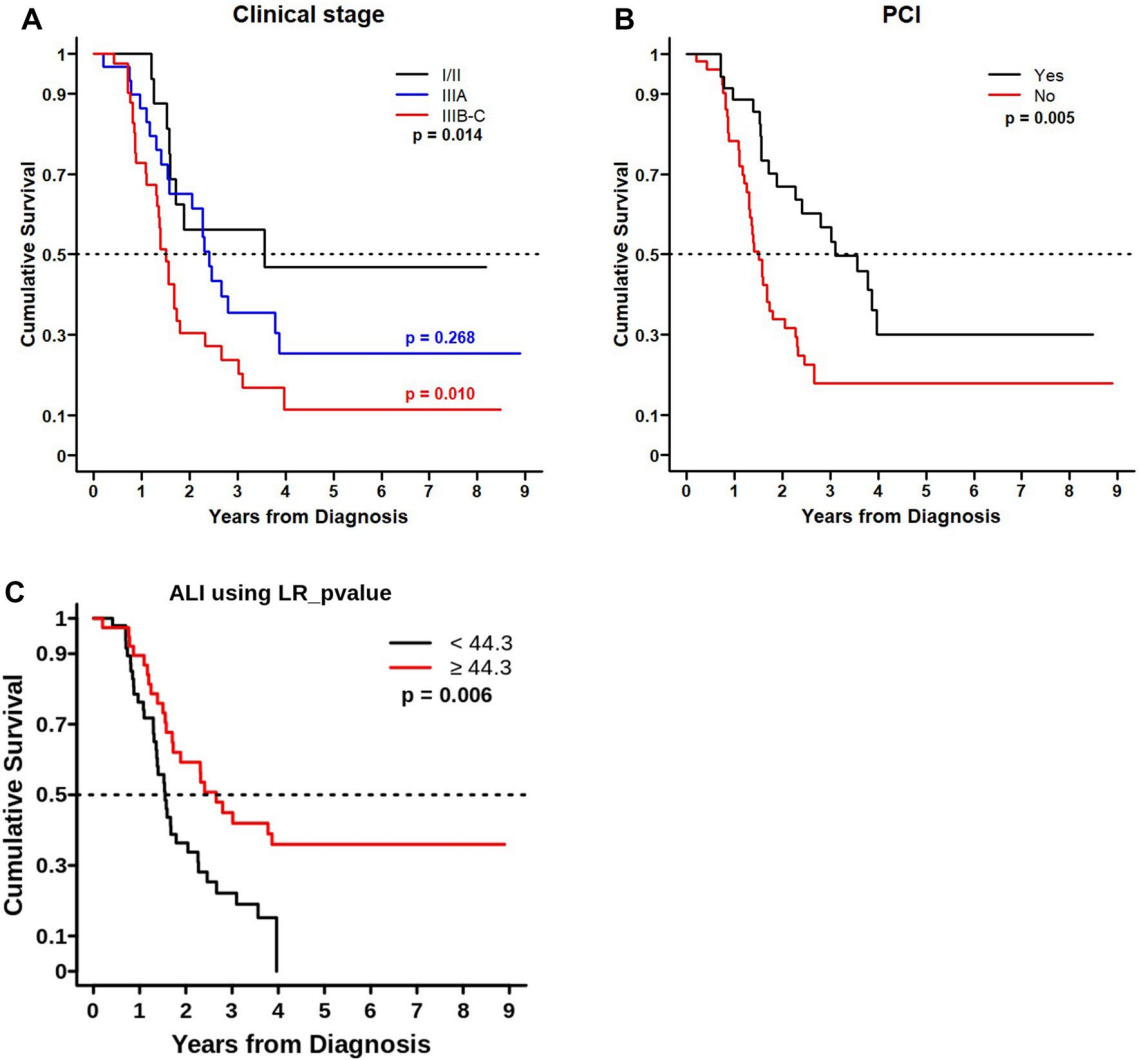
Kaplan–Meier curves for overall survival of patients with limited-stage small-cell lung cancer based on clinical stage, prophylactic cranial irradiation, and the advanced lung cancer inflammation index.

The median OS of all patients was 21.5 (18.6–31.9) months. Univariate and multivariate analyses of OS were performed for clinical and laboratory factors. Several prognostic factors identified through the univariate analysis included higher radiotherapy dosage (≥ 60 Gy; hazard ratio [HR] = 0.532, 95% confidence interval [CI] 0.32–0.90, P = 0.0185 vs. 45–60 Gy), advanced TNM stage (IIIB/IIIC; HR = 2.786, 95% CI 1.27–6.09, P = 0.0103 vs. stage I/II), the presence of PCI (HR = 0.366, 95% CI 0.20–0.66, P = 0.0008), and high ALI (HR = 0.475, 95% CI 0.27–0.84, P = 0.0098). However, in multivariate analysis, advanced TNM stage of IIIB/IIIC (HR = 2.786, 95% CI 1.27–6.09, P = 0.0103 vs. stage I/II) was related to worse survival, though the difference did not reach statistical significance (HR = 2.117, 95% CI 0.95–4.70, P = 0.0656) (Table 2). Additionally, patients who received PCI (P = 0.005) or had a high ALI score (P = 0.006) exhibited statistically better survival rates (Fig. 2).

**Table 2:** Prognostic factors for OS as determined by multivariate Cox proportional hazards regression model.

Variables	Mortality in LS-SCLC			
	Univariate (N = 87)		Multivariate (N = 86)	
	HR (95% CI)	p-value	HR (95% CI)	P-values
Male sex (vs. female)	0.797 (0.39, 1.62)	0.5306		
Age, years	1.014 (0.98, 1.05)	0.3964		
BMI, kg/m^2^	0.965 (0.89, 1.05)	0.4201		
Smoking, ever-smoker (vs. never-smoker)	1.690 (0.23, 12.25)	0.6035		
Radiotherapy dose (Gy), ≥ 60 (vs. 45 ≤ < 60)	0.532 (0.32, 0.90)	0.0185		
ECOG PS ≥ 1 (vs. 0)	1.273 (0.76, 2.12)	0.3560	1.595 (0.93, 2.73)	0.0878
COPD (vs. none)	0.855 (0.51, 1.43)	0.5537		
Diabetes (vs. none)	1.404 (0.83, 2.37)	0.2050		
Hypertension (vs. none)	0.998 (0.59, 1.68)	0.9954		
Chronic kidney disease (vs. none)	1.873 (0.99, 3.56)	0.0550		
TNM stage (vs. stage I/II)				
Stage IIIA	1.590 (0.70, 3.61)	0.2679	1.328 (0.57, 3.09)	0.5100
Stage IIIB/IIIC	2.786 (1.27, 6.09)	0.0103	2.117 (0.95, 4.70)	0.0656
Schedule of radiotherapy				
Sequential (vs. concurrent)	1.252 (0.62, 2.55)	0.5355		
Regimen (vs. etoposide + cisplatin)				
Etoposide + carboplatin	1.369 (0.74, 2.52)	0.3123		
Others	1.477 (0.46, 4.78)	0.5147		
Prophylactic cranial irradiation (vs. none)	0.455 (0.26, 0.79)	0.0047	0.366 (0.20, 0.66)	0.0008
Consolidation chemotherapy (vs. none)	0.827 (0.49, 1.4)	0.4810		
High ALI ≥ 44.3 (vs. < 44.3)	0.470 (0.27, 0.81)	0.0063	0.475 (0.27, 0.84)	0.0098
High LDH (vs. low)	1.291 (0.75, 2.23)	0.3578		

BMI: body mass index; ECOG PS: Eastern Cooperative Oncology Group Performance Status; COPD: chronic obstructive pulmonary disease; ALI: advanced lung cancer inflammation index; LDH: lactate dehydrogenase.

Within the patients receiving etoposide and cisplatin (EP) regimen, ALI score emerged as an independent prognostic factor in patients with LS-SCLC treated with EP regimen in multivariate analysis. A high ALI value was significantly associated with improved long-term survival outcomes (HR = 0.388, 95% CI 0.20–0.75, P = 0.0045) (Table 3). Among patients with high ALI score, those treated with etoposide and carboplatin (EC) regimen exhibited a higher mortality risk compared to EP regimen, although the difference did not reach statistical significance (HR = 3.211, 95% CI 1.00–10.32, P = 0.0501) according to Cox proportional hazard analysis (Table 4).

**Table 3:** Univariate and multivariate Cox proportional hazards analysis of patients with LS-SCLC treated with etoposide and cisplatin (EP) regimen (n = 60).

Variables	Mortality in LS-SCLC treated with etoposide + cisplatin			
	Univariable (N = 60)		Multivariable (N = 60)	
	HR (95% CI)	P-value	HR (95% CI)	P-value
Male sex (vs. female)	1.010 (0.43, 2.40)	0.9823		
Age, years	1.010 (0.97, 1.06)	0.6623		
BMI, kg/m^2^	0.922 (0.83, 1.03)	0.1480		
Smoking, ever-smoker (vs. never-smoker)	NC			
Radiotherapy dose (Gy), ≥ 60 (vs. 45 ≤ < 60)	0.653 (0.35, 1.22)	0.1807		
ECOG PS ≥ 1 (vs. 0)	1.215 (0.66, 2.25)	0.5357		
COPD (vs. none)	1.081 (0.59, 1.99)	0.8024		
Diabetes (vs. none)	1.543 (0.83, 2.87)	0.1711		
Hypertension (vs. none)	1.113 (0.60, 2.06)	0.7348		
Chronic kidney disease (vs. none)	1.341 (0.52, 3.44)	0.5404		
TNM stage (vs. stage I/II)				
Stage IIIA	1.412 (0.54, 3.68)	0.4800		
Stage IIIB/IIIC	2.724 (1.10, 6.77)	0.0310		
Schedule of radiotherapy				
Sequential (vs. concurrent)	0.666 (0.20, 2.16)	0.4981		
Prophylactic cranial irradiation (vs. none)	0.611 (0.33, 1.15)	0.1261	0.554 (0.29, 1.06)	0.0741
Consolidation chemotherapy (vs. none)	0.908 (0.50, 1.67)	0.7565		
High ALI ≥ 44.3 (vs. < 44.3)	0.417 (0.22, 0.79)	0.0070	0.388 (0.20, 0.75)	0.0045
High LDH (vs. low)	1.299 (0.69, 2.45)	0.4184		

BMI: body mass index; ECOG PS: Eastern Cooperative Oncology Group Performance Status; COPD: chronic obstructive pulmonary disease; ALI: advanced lung cancer inflammation index; LDH: lactate dehydrogenase.

**Table 4:** Univariate and multivariate Cox proportional hazards analysis in LS-SCLC with high ALI (n = 38).

Variables	Mortality in LS-SCLC with high ALI			
	Univariable (N = 38)		Multivariable (N = 38)	
	HR (95% CI)	P-values	HR (95% CI)	P-values
Male sex (vs. female)	1.557 (0.36, 6.65)	0.5500	5.842 (0.93, 36.79)	0.0601
Age, years	1.016 (0.95, 1.08)	0.6290		
BMI, kg/m^2^	0.964 (0.84, 1.1)	0.5904		
Smoking, ever-smoker (vs. never-smoker)	N/A			
Radiotherapy dose (Gy), ≥ 60 (vs. 45 ≤ < 60)	0.742 (0.32, 1.70)	0.4797		
ECOG PS ≥ 1 (vs. 0)	1.140 (0.5, 2.59)	0.7542		
COPD (vs. none)	0.799 (0.35, 1.85)	0.5991		
Diabetes (vs. none)	1.079 (0.44, 2.63)	0.8670		
Hypertension (vs. none)	1.365 (0.6, 3.1)	0.4577		
Chronic kidney disease (vs. none)	1.715 (0.58, 5.06)	0.3288	3.811 (0.97, 15.01)	0.0557
TNM stage (vs. stage I/II)				
Stage IIIA	1.293 (0.42, 3.96)	0.6530	2.615 (0.72, 9.49)	0.1438
Stage IIIB/IIIC	2.401 (0.81, 7.08)	0.1122	5.512 (1.62, 18.78)	0.0064
Schedule of radiotherapy				
Sequential (vs. concurrent)	0.907 (0.27, 3.06)	0.8752		
Regimen (vs. etoposide + cisplatin)				
Etoposide + carboplatin	2.151 (0.78, 5.94)	0.1393	3.211 (1.00, 10.32)	0.0501
Others	N/A			
Prophylactic cranial irradiation (vs. none)	0.681 (0.3, 1.56)	0.3622		
Consolidation chemotherapy (vs. none)	0.857 (0.38, 1.96)	0.7154		
High LDH (vs. low)	0.926 (0.4, 2.15)	0.8581		

BMI: body mass index; ECOG PS: Eastern Cooperative Oncology Group Performance Status; COPD: chronic obstructive pulmonary disease; ALI: advanced lung cancer inflammation index; LDH: lactate dehydrogenase.

**Figure 3:**
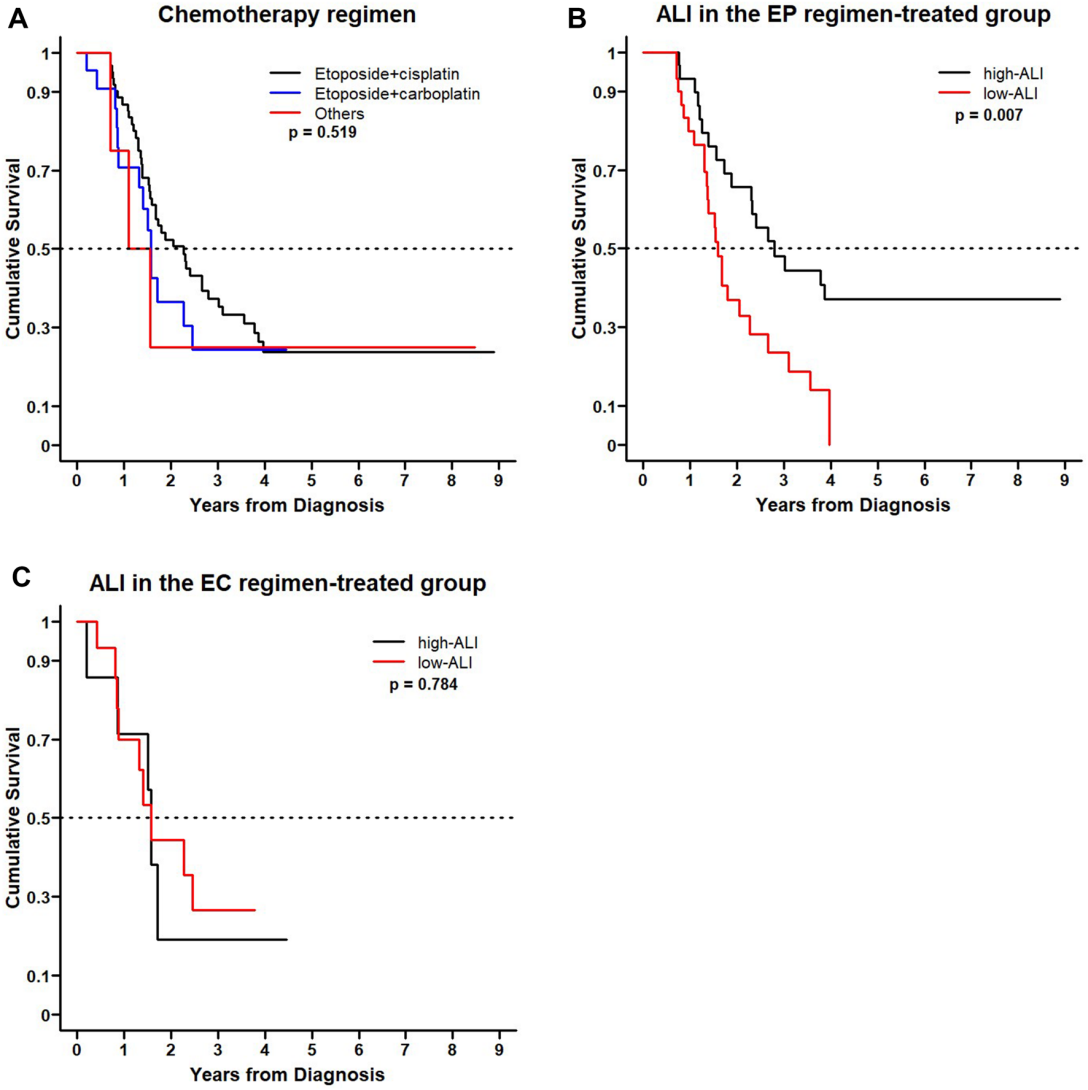
Kaplan–Meier curves for overall survival (OS) of patients with limited-stage small-cell lung cancer treated with etoposide and cisplatin (EP) and etoposide and carboplatin (EC). (**A**) No difference in OS time is observed among chemotherapy regimens (EP, EC and others; P = 0.519). (**B**) In the EP regimen‑treated group, the high ALI group exhibit a significantly longer OS time than that of the low ALI group (P = 0.007). (**C**) In the EC regimen‑treated group, no difference in OS time is observed between the high and low ALI groups (P = 0.784).

The difference in OS was not statistically significant when comparing patients treated with EP to those treated with EC or other regimens (P = 0.519) (Fig. 3A). In subgroup analysis among patients in the EP regimen-treated group, the high-ALI group had a significantly longer OS time compared to the low ALI-group (P = 0.007). However, in the EC regimen-treated group, no difference in OS time was observed between the high and low ALI groups (P = 0.784) (Fig. 3B,C).

## Discussion

In this retrospective study, we established that the pre-treatment ALI serves as a significant prognostic indicator for predicting OS in patients with LS-SCLC undergoing definite CRT. Multivariate analysis revealed that a high ALI score, using a cut-off value of 44.3, was associated with better survival. Furthermore, subgroup analysis within the EP regimen-treated group demonstrated that a high-ALI score was associated with an extended OS time. Interestingly, another subgroup analysis within the high-ALI group suggested that patients receiving the EC regimen may have worse survival compared to those on the EP regimen, although this observation did not achieve statistical significance.

This finding aligns with those of earlier studies, particularly the findings of Jafri et al., who initially developed ALI as a prognostic marker for non-small cell lung cancer (NSCLC), highlighting that a low ALI score (< 18), indicating significant systemic inflammation, was associated with poor progression free survival (PFS) and OS^12^. Similarly, Xiaobo He et al. reported ALI as an independent prognostic marker in SCLC^13^. Following these studies, numerous research has reinforced the prognostic value of various inflammatory biomarkers in the field of lung cancer^17,18^, with NLR and ALI emerging as simple and cost-effective tools for evaluating systemic inflammation. Although not fully understood, the negative correlation of a high NLR with worse OS, observed consistently across various cancer types, may be attributed to the immunological effects of neutrophils and lymphocytes^19^. Neutrophils, through the secretion of tumor growth factors, including vascular endothelial growth factor, IL-6, IL-8, matrix metalloproteinase, and elastase, contribute to the stimulation of the tumor microenvironment, while also suppressing the cytotoxic activity of lymphocytes, activated T cells, and natural killer cells, which are crucial elements in the prognosis and cytotoxic treatment of cancer patients^20–22^. Conversely, lymphocytes play a fundamental role in cytotoxic cell death and cytokine production in tumor cell destruction, making lymphocyte count a recognized predictor of survival in patients with advanced cancer treated with chemotherapy^23,24^. This immunologic effect is heightened in radiotherapy, where radiation induces tumor cell death by directly inducing DNA damage, producing tumor-associated antigens, enhancing antigen presentation, and increasing immune cell infiltration^25,26^. While the prognostic value of inflammatory indices in radiotherapy is documented in multiple studies^27,28^, previous research has predominantly focused on NSCLC or advanced stages treated with systemic chemotherapy. Although some recent studies reported the use of NLR as a prognostic value in NSCLC patients treated with CCRT^29–31^, patients with LS-SCLC were overlooked due to the limited number of patients. Our study exclusively focuses on patients with LS-SCLC treated with definite chemo-radiotherapy, broadening the utility of ALI as a prognostic factor.

An etoposide and platinum combination regimen is the first choice for SCLC treatment, and in multiple randomized controlled trials, the combination of carboplatin or cisplatin with etoposide resulted in no significant difference in PFS or OS^32,33^, making the choice of treatment challenging. Pan et al. initially proposed the potential consideration of pre-treatment NLR for optimizing the choice of treatment in SCLC. Their findings suggested that patients with high NLR exhibited significantly longer PFS when treated with the EP regimen, than that of patients treated with the EC regimen^34^. Liu et al. reported contradicting results, as in the low-NLR group, the treatment with the EP regimen resulted in better OS time than that of the EC regimen treatment, and in the high-NLR group, the EC regimen treatment conferred better OS outcomes than that of the EP regimen treatment^35^. This observation is in line with the results of our study, which incorporated the ALI score, supporting the notion that inflammatory biomarkers can play a role in the selection of chemotherapy regimens in patients with LS-SCLC.

As the understanding of lung cancer biology and the inflammatory effects of cancer development advances, the introduction of immune checkpoint inhibitors (ICIs) has shifted the treatment paradigm. For patients without driver mutation, ICIs such as PD-1 inhibitors are now integral in the treatment of NSCLC^36^. In SCLC, ICIs conventionally have been limited to extensive-stage cases due to the lack of correlating immune biomarkers for treatment response, making patient selection difficult^37,38^. However, recent investigations have explored expanding the role of ICIs to LS-SCLC. Welsh et al. presented a phase I/II study of concurrent pembrolizumab combining CRT in LS-SCLC, showing favorable outcome^39^. Such promising results emphasizes the need for development of prognostic biomarkers. Although PD-L1 expression is recommended biomarker in NSCLC by National Comprehensive Cancer Network (NCCN) guideline, it has shown insufficient in SCLC patients^40^. Inflammatory biomarkers drawn from peripheral blood, such as NLR and/or PLR are showing prognostic value in patients treated with ICIs in NSCLC, as low NLR and PLR are associated with better survival outcomes^41–43^. Shiroyama et al. first demonstrated the relationship between ALI and the survival outcome of NSCLC patients treated with immunotherapy, as a low ALI score is associated with poor prognosis and patients may benefit from continued nivolumab treatment^44^. Mountzio et al. conducted a large comparative study showing the prognostic value of ALI for patients with advanced NSCLC treated with PD-L1 inhibitors alone^45^. As shown by our study, ALI may help broaden patient selection in patients with LS-SCLC for immunotherapy; however, further investigation is needed.

Despite these insights, out study has several limitations. The retrospective design and a relatively small number of patients constrain generalization and introduce selection bias. The cut-off value for defining high and low ALI groups needs further validation. As previously mentioned, Jafri et al. drew cutoff value at 18 which was around 50th percentile mark to divide high and low ALI group^12^. In study of prognostic value of ALI in SCLC following surgical resection, Hu et al. drew cut off value at 48.2 using ROC curve analysis^15^. As such, standardizing program for ALI and its cutoff value needs further discussion. Furthermore, other inflammatory blood parameters such as CRP, monocyte, platelet, hemoglobin counts are not included in calculating ALI. As previous studies have shown prognostic value of lymphocyte-to-CRP ratio (LCR), systemic immune-inflammation index (SII), systemic inflammation response index (SIRI), and hemoglobin-to-red-cell-distribution-width ratio (HRR), further investigation is needed to supplement the ALI formula^46^. Additionally, our study did not investigate baseline conditions that could affect NLR such as sepsis, pneumonia or cardiovascular disease due to limited data. Therefore, we are unable to provide additional information on this aspect.

In conclusion, this study is the first to indicate the potential use of ALI values for predicting survival in patients with LS-SCLC undergoing definite CRT. Moreover, our data imply that pre-treatment ALI score, as a blood-based biomarker, could be useful to identify specific subgroup of LS-SCLC likely to benefit from definite CRT. Additionally, it offers valuable guidance for selecting appropriate chemotherapy regimen in those patients, providing a basis for further investigations.

## Methods

### Study population and data collection

A retrospective study was conducted including patients with LS-SCLC treated with definite CRT at four university-affiliated hospitals in South Korea: Hanyang University Hospital, Hanyang Guri University Hospital, Kyeonghi University Hospital, and Kangdong Kyeonghi University hospital, spanning from January 2005 to December 2019. Inclusion criteria comprised a pathologically proven diagnosis of SCLC, confirmed as LS-SCLC (confined to one hemithorax, mediastinum, contralateral hilus, and supraclavicular regions), and completion of combined chemotherapy and TRT as planned. Patients confirmed as having extensive stage small-cell lung cancer were excluded.

The study included 87 patients with LS-SCLC, and survival was defined as period from the date of diagnosis to death from any other cause or the last follow-up.

Data on demographic factors associated with mortality were collected, including sex, body mass index (BMI), age at the time of SCLC diagnosis, calendar year of SCLC diagnosis, smoking status, years of initiation for radiotherapy, radiation dosage, PS, underlying diseases including CKD, TNM stage for lung cancer, schedule of radiotherapy, chemotherapy regimen, status of PCI, and consolidation chemotherapy. Pretreatment blood samples were collected for white blood cell counts, absolute lymphocyte counts, absolute neutrophil counts, platelet counts, serum albumin, LDH, and CRP.

ALI, proposed as a potential prognostic biomarker, was calculated using the formula: BMI × serum albumin/NLR, with NLR representing the proportion of neutrophil counts to lymphocyte counts in peripheral blood.

Patients eligible for the study underwent baseline staging computed tomography, irradiation of all pathological upon completion of TRT, and at least one course of chemotherapy. All enrolled patients received thoracic radiotherapy of at least 45 Gy in once-daily doses of 2 Gy administered over 6 weeks using involved-field TRT. Administration of PCI was optional for those responding well to CRT, and the decision to add consolidation chemotherapy was made individually, considering the patient’s preference and performance status.

### Statistical analysis

SAS software (version 9.4; SAS Institute, Cary, NC, USA) was used for patient matching, and R software (version 3.4.2; R Development Core Team, Vienna, Austria) for all other statistical analyses, following an assessment of the data distribution’s normality using the Shapiro–Wilk test. Continuous variables were compared using the Student’s t-test or Mann–Whitney U test, and categorical variables using the χ2 or Fisher’s exact tests. Nominal variables are expressed as frequencies and percentages, while continuous variables are presented as means ± SD if normally distributed, and as medians with quartile range if non-normally distributed. Follow-up began at the time of cancer diagnosis and ended at the time of death or the last follow-up visit for patients without an event (whichever occurred first).

Survival curves were estimated using the Kaplan–Meier method, and differences in survival among groups were assessed using a two-sided log-rank test. Univariate associations of mortality with clinicopathological variables of interest were analyzed using a Cox proportional hazards regression model. The best subset selection method was then applied to build multivariate models, including variables significant in the univariate analysis, using the backward stepwise selection method. The Akaike information criterion (AIC) was computed to select the model with the smallest AIC value.

The years of treatment initiation were categorized into 2005–2009, 2010–2014, and 2015–2019, with a difference in radiation dose in 2015–2019 compared to 2005–2014 (mean 62.02 vs 51.69 Gy). Although the optimal radiation dose and schedule has not been established, it is widely recognized that increasing radiation dose is associate with better survival outcomes^47,48^. Therefore, we used radiation dose rather than years of initiation for radiotherapy as a variable in the final multivariable analysis.

The prognostic value of ALI was determined using the Biostatistical Tool, Cutoff Finder, to obtain the cut-off point. Subgroup analysis was performed for the low-ALI (ALI < 44.3) and high‑ALI (ALI ≥ 44.3) groups using Cox proportional hazards regression analysis, estimating HRs and calculating P-values for various prognostic factors. Additional subgroup analysis based on the chemotherapy regimen provided to patients was conducted, considering previous research showing relevant yet contradicting results with NLR and EP, EC regimens^34,35^. Thus, we hypothesized that ALI could be a relevant prognostic factor in selecting a chemotherapy-regimen.

Survival curves were generated using the Kaplan–Meier method. All tests were two-sided, and P-values < 0.05 were considered statistically significant.

### Ethics statement

The study protocol received approval from the Institutional Review Board (IRB) of Hanyang University Hospital, Seoul, South Korea (IRB No.–2023-01-002), in accordance with the principles outlined in the Declaration of Helsinki. All data were anonymized before analysis, and the IRB at Hanyang University Hospital waived the requirement for informed consent from the study participants due to the retrospective nature of the study.

## Data Availability

The datasets generated during and/or analyzed during the current study are available from the corresponding author on reasonable request.
